# Microencapsulation of *Vitis vinifera* grape pulp phenolic extract using maltodextrin and its application in gummy candy enrichment

**DOI:** 10.1002/fsn3.4005

**Published:** 2024-02-13

**Authors:** Maryam Heidari, Akram Pezeshki, Babak Ghanbarzadeh, Hamed Hamishehkar, Farnaz Ahmadzadeh Nobari Azar, Maryam Mohammadi, Marjan Ghorbani

**Affiliations:** ^1^ Department of Food Science and Technology, Faculty of Agriculture University of Tabriz Tabriz Iran; ^2^ Department of Food Engineering, Faculty of Engineering Near East University Nicosia Cyprus; ^3^ Drug Applied Research Center Tabriz University of Medical Sciences Tabriz Iran; ^4^ Department of Food Science and Engineering, Faculty of Agriculture University of Kurdistan Sanandaj Iran; ^5^ Nutrition Research Center Tabriz University of Medical Sciences Tabriz Iran

**Keywords:** antioxidant activity, green extraction, gummy candy, microencapsulation

## Abstract

Phenolic extract of *Vitis vinifera* grape pulp was prepared using ultrasonication at frequencies of 28, 40, and 28–40 kHz with a 1:10, 1:15, and 1:20 ratio of solid (grape pulp) to water. The 1:10 ratio and 40 kHz frequency were considered optimal conditions for the preparation of red grape pulp extract (RGPE). Then, RGPE was encapsulated within maltodextrin using a spray drying technique, and the produced powder was used in the gummy candy production. The results revealed that the phenolic content of the powder was almost constant during the storage time. The solubility of the powder decreased, whereas its moisture content increased during the 45‐day storage period. The results of scanning electron microscopy showed that the produced microparticles had spherical shapes with a micron size. Fourier‐transform infrared spectroscopy and X‐ray diffraction showed that RGPE was encapsulated in the structure of maltodextrin through the formation of hydrogen bonds, considering the amorphous structure of the powder. The antioxidant properties of the microencapsulated RGPE containing gummy candy were preserved. Sensory evaluation and colorimetric values of the enriched gummy candy had acceptable results compared to the control sample. In general, microencapsulation of RGPE within maltodextrin as a carrier using the spray drying technique and its application in gummy candy enrichment could be useful.

## Graphical abstract

Phenolic extract of *Vitis vinifera* grape pulp was prepared using ultrasonication at frequencies of 28, 40, and 28–40 kHz with a 1:10, 1:15, and 1:20 ratio of solid (grape pulp) to water. The 1:10 ratio and 40 kHz frequency were considered optimal conditions for the preparation of red grape pulp extract (RGPE). Then, RGPE was encapsulated within maltodextrin using the spray drying technique, and the produced powder was used in the gummy candy production. The results revealed that the phenolic content of the powder was almost constant during the storage time. The solubility of the powder decreased, whereas its moisture content increased during storage period of 45 days. The results of scanning electron microscopy showed that the produced microparticles had spherical shapes with a micron size.

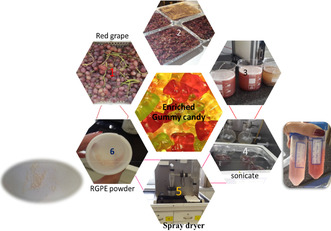

## 1 INTRODUCTION

The vine with the scientific name (*Vinifera*) belongs to the *Vitaceae* family. There are about 11 genera and more than 600 species in this family. The most important genus in this family is the grape. The antioxidant activity of red grapes is related to their polyphenolic content (Hasan et al., 2014). Resveratrol (trans‐3,5,4′‐trihydroxystilbene) is one of the plant polyphenols of grape, which belongs to the stilbenes' family. Due to having two aromatic groups, resveratrol acts as an antioxidant, preventing oxidative stress and therefore the progression of atherosclerosis (Athar et al., 2009). Grape pulp, the inedible part of the grape obtained from the grape juice extraction process, is usually considered animal feed. Due to the high nutritional properties of grape pulp, it is necessary to extract it and use it in food fortification. In recent years, the implementation of ultrasound‐assisted extraction as a green technology has reduced environmental pollution. Also, several techniques, such as microencapsulation or encapsulation, have been used to protect bioactive compounds due to their sensitivity to environmental conditions (light, oxygen, and temperature; Rahimipanah et al., 2011). Among the various microencapsulation methods, spray drying is the most widely used method in the food industry. This method is highly recommended for microencapsulation of sensitive compounds such as polyphenols due to rapid solvent evaporation, leading to limited heat exposure time (Zhang et al., 2020). Maltodextrin is one of the biopolymers widely used for spray drying due to its relatively low price, neutral taste, and low viscosity (Wang et al., 2013). This study was aimed at extracting the phenolic and antioxidant compounds of red grape pulp using a green extraction method (ultrasound‐assisted extraction). Then, the produced extract was microencapsulated within maltodextrin using a spray dryer to be used in the enrichment of gummy candy as a source of antioxidants as well as natural pigments.

## 2 MATERIALS AND METHODS

### 2.1 Materials

Red grape pulp was purchased from Takdaneh Co. (Marand, Iran). Gelatin was provided by Farmand Co. (Iran). Maltodextrin (DE = 20) was obtained from (Merck, Germany). Sodium carbonate was purchased from Hamon Teb Markazi Co. (Iran). 2,2‐Diphenyl‐1‐picrylhydrazyl (DPPH) was provided by Sigma Aldrich Co. (St. Louis, MO, USA). Glucose syrup and sugar were obtained from Shirin Asal Co. (Iran). Folin–Ciocalteu reagent, potassium acetate, and aluminum chloride were obtained from Merck Co. (Darmstadt, HE, Germany). Methanol was purchased from Dr. Mojallali Industrial Chemical Complex Co. (Iran).

### 2.2 Production of red grape pulp extract

To extract phenolic compounds, a mixture of grape pulp and distilled water was prepared in ratios of 1:10, 1:15, and 1:20, respectively. The resulting mixture was placed in an ultrasonic bath at 50°C and frequencies of 28, 40, and 28–40 kHz for 25 min. Then, the samples were placed on a magnetic stirrer at 25°C and 283 g for 20 min and finally passed through Whatman Grade 42 filter paper to separate the impurities. For the best separation of the remaining impurities, the samples were centrifuged at 241 g for 20 min. The obtained extracts were stored at 4°C for further experiments.

### 2.3 Production of powder

#### 2.3.1 Preparation of liquid feed

After performing grape pulp extract tests, the extract with the highest amount of phenolic, flavonoid, and antioxidant compounds was prepared for spray drying. To prepare a 30% w/v solution of maltodextrin as spray dryer feed, 30 g of maltodextrin was dissolved in 100 mL of extract. For complete hydration, the solution was stirred on a low‐speed magnetic stirrer for about 24 h. Finally, the samples were ready for the spray drying process.

#### 2.3.2 Spray drying process

The laboratory spray dryer (Dorsa Beh Saz, Iran) was used for the drying process. The operating conditions were inlet temperature of 120°C; outlet temperature of 80°C; feed rate of 3 mL/min; atomization airflow of 10 L/h; drying airspeed of 2.50 m/s; and atomizing nozzle diameter of 1.2 mm.

The produced powders were kept inside the glass desiccator until they reached a constant temperature and moisture content. Placement in the desiccator was to prevent moisture absorption of the produced powders and ensure physical stability and active compound preservation, not their final drying. The moisture content of the powder after spray drying was 4.8. The coated powder was then stored in dark glass away from the light to measure the percentage of phenolic compounds and antioxidant capacity for 45 days.

### 2.4 Physicochemical characterization

#### 2.4.1 Measurement of total phenolic and flavonoid content and antioxidant capacity

First, 2 mL of methanol, acetic acid, and distilled water in the ratios of 50/8/50 V/V/V were added to 200 mg of microencapsulated red grape pulp extract (RGPE) and stirred for 1 min. Next, the mixture was subjected to ultrasound for 20 min in two stages (with 100% intensity and a frequency of 20 kHz), and finally it was centrifuged at 8365  g for 10 min. The supernatant was used to measure phenols, flavonoids, and antioxidant capacity.

The amount of total phenol was measured using Singelton and Rossi (1965) method, with some modifications. The control sample was a methanolic mixture without extract (1 mL of the distilled water was mixed with 1 mL of 80% methanol, and the methanolic mixture was prepared). Then, 500 μL of distilled water, 125 μL of 10% folin ciocalteu regent, and 1.25 mL of 7.5% sodium carbonate were added to 125 μL of the prepared methanolic mixture, and the volume was brought to 3 mL with distilled water. In samples, 1 mL of the samples (extract and maltodextrin powder containing extract) was mixed with 1 mL of 80% methanol, and the methanolic mixture was prepared. After incubation in the dark for 90 min at room temperature (25°C), the sample absorbance was read at 765 nm. The standard curve (*R* ^2^ = .979) was plotted using different concentrations of gallic acid and adsorption measurements at 765 nm. The total phenolic content was expressed in mg of gallic acid equivalent per liter of sample. The control sample consisted of all compounds except the extract.

Flavonoid content was measured using the colorimetric method. First, 1.5 mL of 80% methanol was added to 5 mL of the sample (RGPE and maltodextrin powder containing RGPE). Next, 100 μL of 10% aluminum chloride and 100 μL of potassium acetate were added to the prepared methanolic mixture. Then, 2.8 mL of distilled water was added to the mixture and kept in the dark for 30 min. Finally, the absorbance was measured at 415 nm, and the total flavonoid content was reported in mg of quercetin per gram of sample. The control sample included all compounds except the extract (Kim et al., 2002).

The ability of free radical scavenging of the samples (RGPE and maltodextrin powder containing RGPE) was determined by the method of Brand‐Williams et al. (1995), with some modifications. For this purpose, 1 mL of the sample and 1 mL of 80% methanol were mixed and allowed to stand for 24 h. Then, 1.95 mL of 0.04 mM DPPH was added to 50 μL of the methanolic mixture, and finally, the absorbance was read at 517 nm after 30 min of sample rest.

#### 2.4.2 Scanning electron microscopy (SEM)

The surface morphology of samples was investigated using a scanning electron microscope (MIRA3 FEG‐SEM, Check). The samples were diluted with DDW, dispersed on the lamel, and placed in an oven to be dried. The dehydrated samples were then coated with gold in a low‐vacuum coater (EM ACE200, Leica, Wetzlar, Germany) at a deposition rate of 0.51 Å s^−1^ for 180 s, using 3–5 mA of current at a pressure of 0.2 Pa. SEM images of coated samples were obtained at an accelerating voltage of 15 kV, and the morphology of particles was observed. (Santhalakshmy et al., 2015).

#### 2.4.3 Fourier‐transform infrared (FTIR) spectroscopy

The FTIR patterns were recorded by an FTIR device (8400 IS, Shimadzu, Tokyo, Japan). To prepare the samples, first the pure maltodextrin and the maltodextrin powder containing RGPE were mixed with potassium bromide (KBr) in a ratio of 1:100, and analysis was done in the wavenumber range of 4000–500 cm^−1^ (Pezeshki et al., 2019).

#### 2.4.4 X‐ray diffraction (XRD)

XRD was performed using an X‐ray diffraction device (D500, Siemens, Berlin, Germany). To perform this test, samples were exposed to an X‐ray generator with a voltage of 40 kW, a filament current of 30 mA, and a wavelength of 1.54 Å. The reflected radiation was collected at 25°C, and a diagram of its reflection intensity was drawn. The test speed was 1°/min, and the step size was 0.05 (Ahmadzade Nobari Azar et al., 2021).

#### 2.4.5 Measurement of powder production efficiency

Powder production efficiency was measured by calculating the ratio of the mass percentage of powder obtained to the total mass of solids in the feed (in terms of dry matter) using Equation 1 (Sarabandi et al., 2014):

(1) $$\text{Powder production efficiency}\;\left(\%\right)=\frac{\text{The mass of the final product}}{\text{The total mass of feed material}}\times 100$$

#### 2.4.6 Measurement of the moisture content of the powder

To measure the moisture content of the powder, the washed plate was placed in the oven at 105°C for 20 min and then in a desiccator to reach room temperature. Next, 0.2 g of the powder was added to the plate and placed in an oven at 105°C for 2 h. After cooling and reaching a constant weight in the desiccator, the sample was weighed, and the moisture content of the powder (%) was calculated using Equation 2 (Atomizer, 1978):

(2) $$\text{Moisture content}\;\left(\%\right)=\frac{\text{Total weight of powder and container}-\text{Weight of dried powder}}{\text{Total weight of powder and container}-\text{Empty container weight}}$$

#### 2.4.7 Powder solubility

To measure the solubility index of the powder, 100 mL of water was added to 1 g of the sample and placed on a magnetic stirrer at 16435 g for 4 min. It was then centrifuged at 3000 × g for 4 min. Finally, 25 mL of the supernatant solution was placed in an oven at 105°C for 5 h. The degree of solubility in water (%) was calculated using Equation 3 (Cano‐Chauca et al., 2005):

(3) $$\text{Solubility}=\frac{\text{Weight of the powder in the supernatant}\;\left(g\right)\;}{\text{Weight of the powder in the solution}\;\left(g\right)\;}\times 100$$

#### 2.4.8 Gummy candy production

To produce gummy candy, the sugar (30.17% w/w) and glucose syrup (30.17% w/w) were dissolved in lukewarm water. Next, the gelatin (19.45% w/w) was dissolved in this mixture under agitation using a magnetic stirrer with the help of indirect heat and allowed to reach ambient temperature. The soluble solids content of the extract used for the maltodextrin‐extract feed solution was 2.5% w/v, then the RGPE powder (5% w/w) and citric acid (3% w/w) were added to the mixture, poured into the pre‐greased molds, and kept at 4°C for 2 h. Finally, it was taken out of the mold and stored at 4°C for further experiments (Charoen et al., 2015).

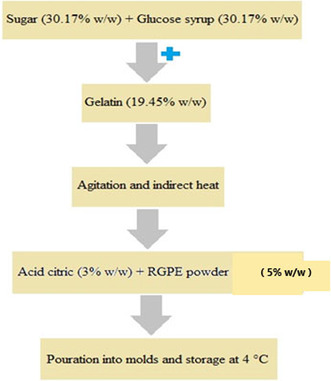

### 2.5 Measurement of the antioxidant activity of gummy candy

To perform the experiment, 3 g of the produced gummy candy was dissolved in 3 mL of distilled water and then placed in an ultrasonic bath for 30 min. After the gummy candy became liquid, it was centrifuged at 7000 rpm for 10 min. Finally, according to the above‐mentioned methods, 50 μL of the supernatant was added to 1.95 mL of methanolic DPPH (0.04 mM). After incubation in the dark at 25°C for 30 min, the absorbance of the samples was read at 517 nm. A gummy candy sample without RGPE powder was considered as a control sample (Rodríguez‐Sánchez et al., 2017).

#### 2.5.1 Color analysis

The digital imaging method was used to analyze the color of the samples. The samples were placed in a Hunter lab simulated chamber with a white wall (50 × 50 × 50) and two fluorescent lamps with a white light inside and a perfectly uniform light distribution. Imaging was done with a 20‐megapixel digital camera (Sony DSC‐W830) perpendicular to the sample (30 cm apart). The measurement criterion was the white color of the paper. The obtained images were transferred to the Photoshop software (Adobe Photoshop CC2015), and their color parameters (*L⃰*, *a⃰*, and *b⃰*) were obtained. The chroma (*C⃰*) index and the hue angle were calculated using Equations 4 and 5 (Ghasemlou et al., 2011):

(4) $$\text{Chroma}=\sqrt{{\left(a\right)}^{2}+\left(b\right)2}$$

(5) $$\mathrm{Hue}=\tan^{-1}\left(b/a\right)$$

#### 2.5.2 Sensory evaluation

Sensory evaluation for taste, odor, color, and general acceptance was performed by 10 semi‐trained sensory evaluators using a 5‐point hedonic test. The panelists were selected from the 30 female and male bachelor of science (B.S) students from the department of food science and technology (university of Tabriz, Iran). They were in the range of 18–22 years old, and before the sensory analysis, they visited the gummy candy production line (Aydin factory, Tabriz, Iran) to become aware of the sensory characteristics of gummy candy and were trained to some extent to do sensory analysis (semi‐trained panelists). Finally, 10 of them (six female and four male) were selected for the analysis of gummy candy under evaluation tests. Sensory evaluation of test samples (5 g) randomly selected in gummy candy wrappers at ambient temperature (23°C) was done by the panel members. The quality scale ranges from poor to excellent quality, and the intensity scale ranges from low to high intensity for off‐flavor, sweet taste, astringency, and gumminess attributes. A score of 1 was considered unacceptable, 2 for relatively satisfactory, 3 for satisfactory, 4 for good, and 5 for excellent quality. The analysis of data was done by analysis of variance using a completely random block design in SPPS 26. The comparison of means was done by the Duncan test at a 5% significance level (Rodríguez‐Sánchez et al., 2017).

#### 2.5.3 Water activity

The water activity of the samples was measured using the AquaLab (Pullman WA, USA) apparatus, which was adjusted at room temperature, varying from 20 to 25°C.

AquaLab uses the chilled‐mirror dew point technique to measure the water activity of a sample. In an instrument that uses the dew point technique, the sample is equilibrated with the headspace of a sealed chamber that contains a mirror and a means of detecting condensation on the mirror.

Water activity was measured according to the National Standard of Iran (No. 2682) (ISIRI, 2017).

### 2.6 Statistical analysis

The results were analyzed based on a completely randomized block design during 45 days of storage with SPSS software to calculate the significance of the samples compared to the control sample and *p*‐value. Duncan's multi‐domain test at the 5% probability level was used to compare the means.

## 3 RESULTS AND DISCUSSION

### 3.1 The effect of the solid (grape pulp) to water ratio on the extraction of phenolic and flavonoid compounds

**FIGURE 1:**
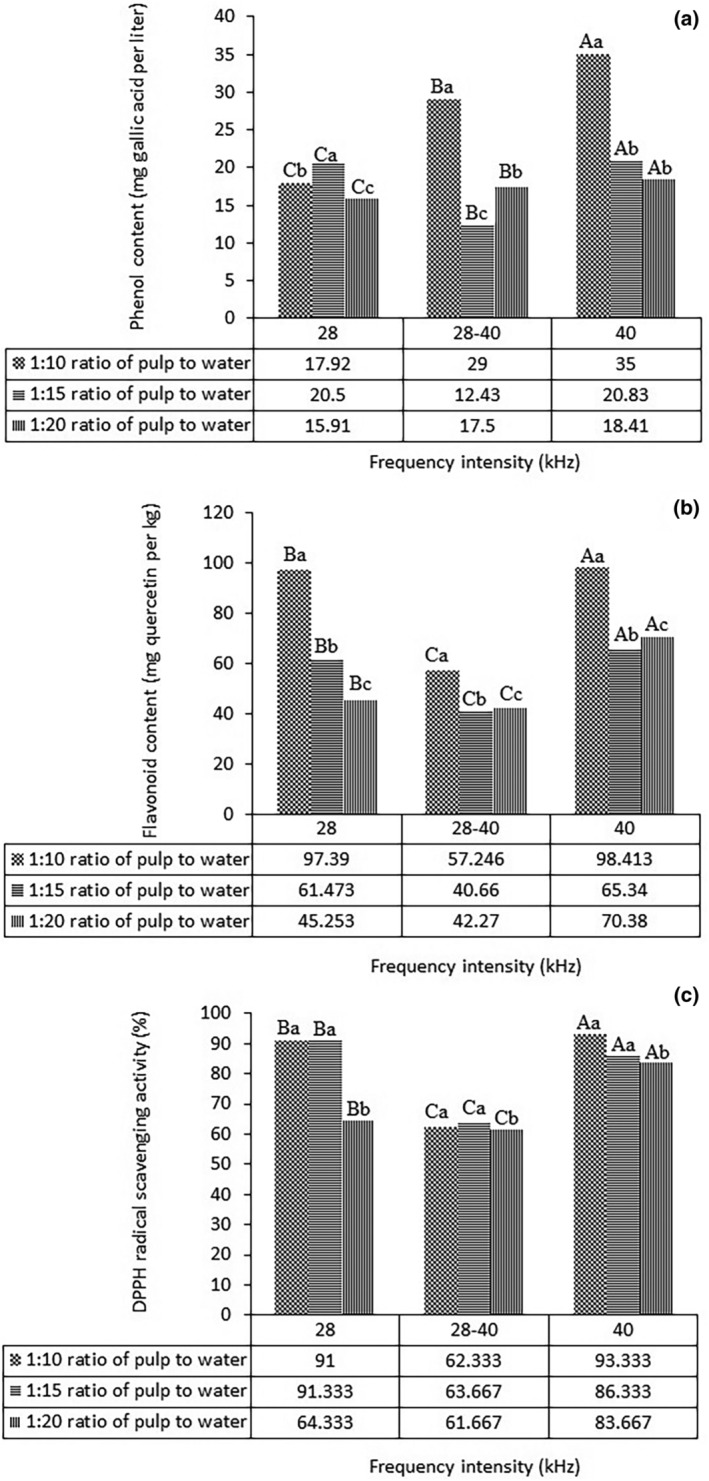
(a) Phenol (mg gallic acid per liter), (b) flavonoid (mg quercetin per kg) content, and (c) DPPH free radical scavenging activity (%) of red grape pulp extract (RGPE) in different ratios of dry matter (grape pulp) to water (Uppercase letters indicate a significant difference of 5% between frequencies, and lowercase letters indicate a significant difference of 5% between different ratios of dry matter to water).

Figure 1a,b shows the effect of different ratios of dry matter (pulp) to water and frequencies on the extraction of phenolic and flavonoid compounds from red grape pulp, respectively. The use of a 1:10 ratio of pulp to water and 40 kHz frequency intensity resulted in the highest amount of phenolic and flavonoid compounds. The reason for the increase in the extraction of phenolic and flavonoid compounds with the increase in ultrasound frequency could be due to the further destruction of the cell wall of grape pulp and also the connections between phenolic compounds and other components (polysaccharides and proteins). As a result, the solubility of phenolic compounds as well as the effect of mass transfer are improved, and the extraction rate of compounds is increased (Chemat & Khan, 2011). Bhat et al. (2011) reported that the number of phenolic compounds in lemon juice increased with the sonication process. When the dry matter is exposed to a large amount of solvent, the solvent easily penetrates the dry matter and dissolves more of it, affecting the extraction efficiency. The higher the ratio of water to dry matter, the longer it takes to reach concentration equilibrium, and ultimately, the higher the concentration efficiency. Alighourchi et al. (2013), about the extraction of phenolic compounds from pomegranate juice using ultrasound, reported that the number of phenolic compounds increased by increasing the reaction time and the sonication frequency.

### 3.2 The effect of solid (grape pulp) to water ratio and frequency intensity on antioxidant activity

As shown in Figure 1c, the use of a ratio of 1:10 dry matter to water and a frequency intensity of 40 kHz resulted in the highest antioxidant activity. These parameters were used as the optimal formulation for the preparation of RGPE powder using the spray dryer. Phenolic compounds are one of the most essential compounds for creating antioxidant properties in grapes. Compositions that have a high phenolic content also have higher antioxidant properties. Phenolic compounds can neutralize free radicals and act as hydrogen donors due to their hydroxyl groups, which prevent oxidation and its negative effects. In addition to antioxidant properties, these compounds have anti‐cancer and anti‐viral properties and are considered functional compounds (Bhat et al., 2011). According to the results of phenolic, flavonoid, and antioxidant content measurements of RGPE, the 1:10 ratio and 40 kHz frequency intensity were recognized as optimal conditions for the preparation of an aqueous extract of grape pulp.

### 3.3 Phenolic and antioxidant compounds of the powder during 45 days of storage

**FIGURE 2:**
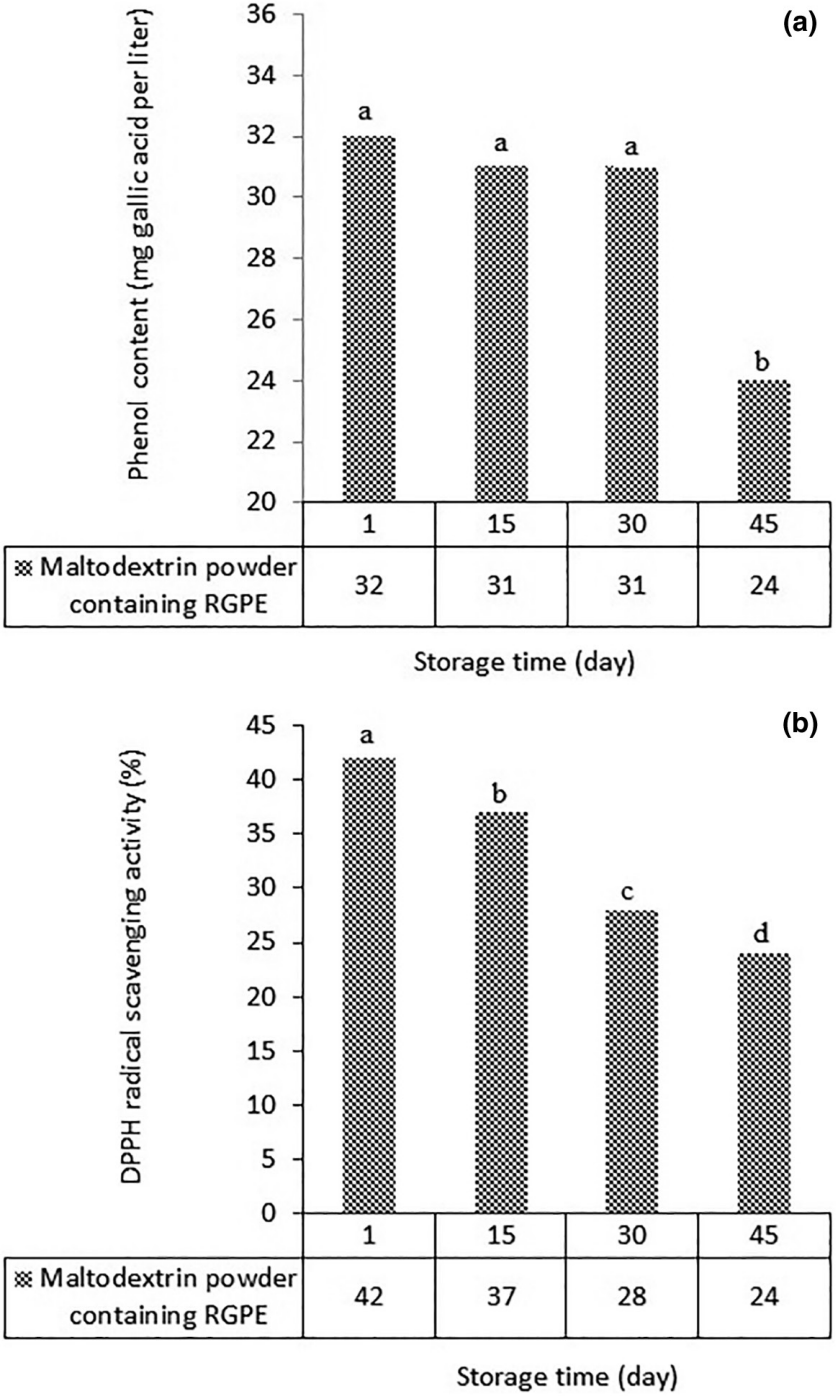
(a) Phenol content (gallic acid per liter), and (b) antioxidant activity (%) of maltodextrin powder containing red grape pulp extract (RGPE) during 45 days of storage.

Dryer temperature is one of the factors affecting the antioxidant properties and phenol content of the final powder. Due to the destructive effect of temperature on phenolic compounds, the amount of phenolic compounds and, consequently, antioxidant activity decreases with increasing temperature. During the storage time, the phenolic content of the powder was almost constant until day 30 and then decreased until day 45 (Figure 2a). The higher antioxidant property of the powder in comparison with the RGPE (Figure 2b) is related to the increase in the concentration of the phenolic content in the experimental sample to measure the phenolic content. As the dryer temperature increased, the antioxidant properties of the powder decreased, which was attributed to the effect of the high dryer temperature on the destruction of phenolic compounds. The rate of feed flow into the dryer chamber also affects the antioxidant properties. The slower the feed flow rate, the longer the exposure time to high temperatures inside the chamber. Therefore, the phenolic compounds and antioxidant properties of RGPE are reduced. Kha (2010) reached similar conclusions about gac fruit powder. They found that the reduction in antioxidant properties at high temperatures could be attributed to the adverse effect of temperature on phenolic compounds and their destruction at high temperatures.

### 3.4 Microstructure of maltodextrin powder containing RGPE: SEM

**FIGURE 3:**
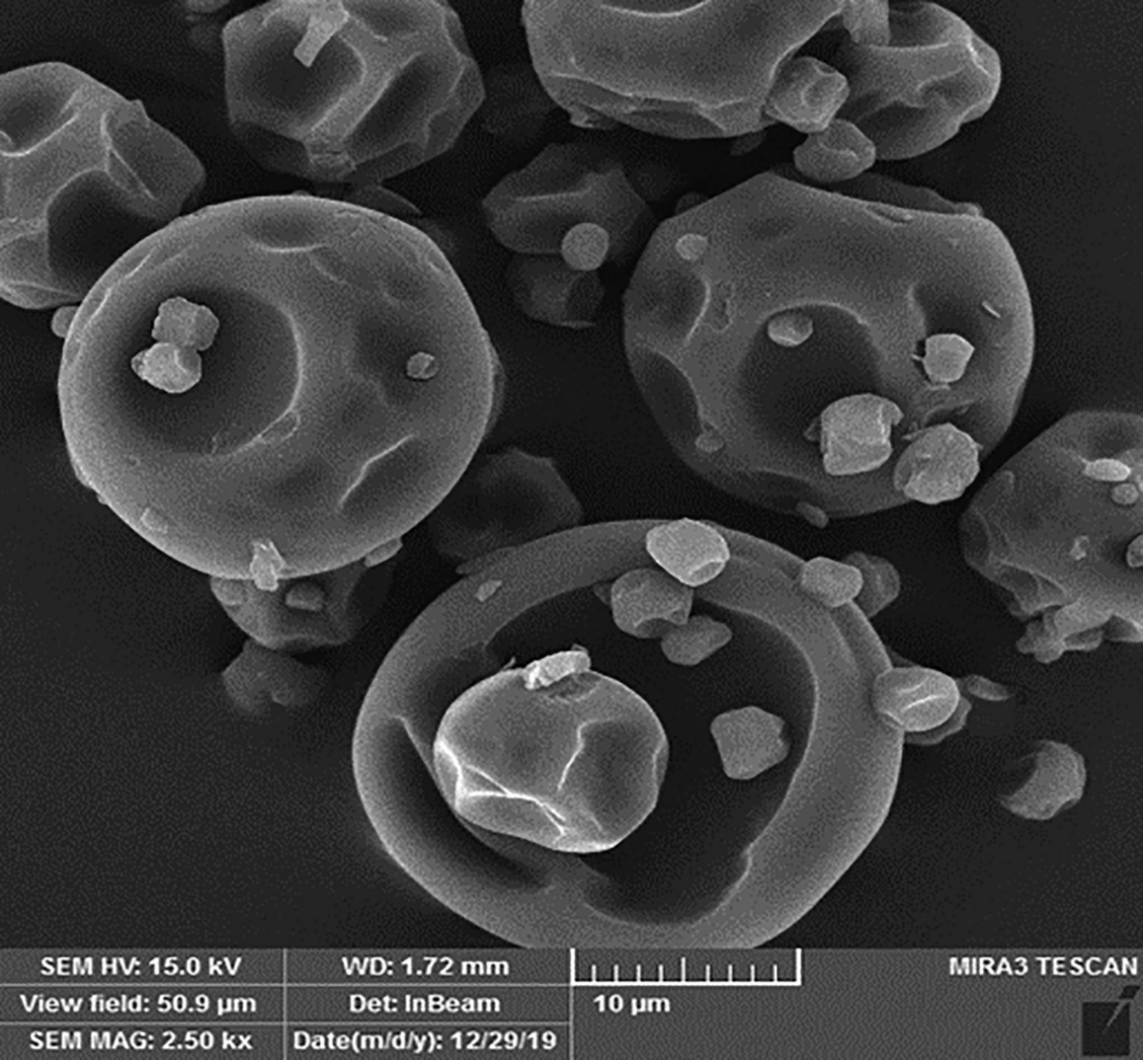
Morphology of maltodextrin powder containing red grape pulp extract (RGPE) obtained from SEM.

SEM was done to obtain accurate information on the size and shape of the maltodextrin powder containing RGPE. Figure 3 shows the particle structure of the powder containing RGPE (various spherical and irregular shapes). The powder particles were approximately the same size and less than 10 μm in size. Also, a slight shrinkage was evident on the surface of the particles, which could be attributed to the lack of a maltodextrin layer leading to an increase in the rate of moisture removal from the particle surface. The production of particles with irregular and wrinkled surfaces is mainly a common phenomenon in the spray drying of various products. Depending on the type of carrier used, an increase in the rate of moisture evaporation and crust formation produces a wall with a more spherical surface. Also, higher temperatures give less opportunity for the particle wall to wrinkle (Wang et al., 2013). Sarabandi et al. (2019) reported similar results on the effect of spray drying conditions on the physicochemical properties of marjoram extract powder.

### 3.5 FTIR

**FIGURE 4:**
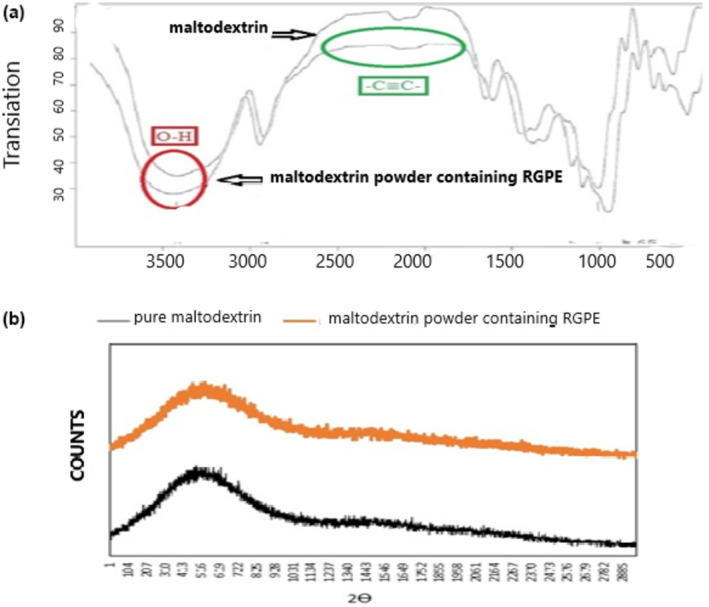
(a) FTIR spectrum and (b) XRD patterns of pure maltodextrin and maltodextrin powder containing red grape pulp extract (RGPE).

Infrared spectroscopy is an effective qualitative test to identify the functional groups present in the structure of complex organic compounds and to show the structural changes in the materials. Each of the functional groups has a specific absorption intensity. The absorption intensity changes with the emergence of a new composite structure and structure alterations. Figure 4a shows the IR spectra of pure maltodextrin and the maltodextrin powder containing RGPE. As is clear, the spectrum of both samples showed an absorption peak in the wavenumber range of 3600–3200 cm^−1^, which is related to the stretching vibrations of the OH groups. The widening of the peak at a wavenumber of 3500 cm^−1^ is probably due to the formation of a bond between maltodextrin and phenolic extract. Other interactions include a decrease in the intensity of the peak appearing in the spectrum of maltodextrin powder containing RGPE at the wave number of 2500–2000 cm^−1^, which is related to the C≡C factor group in alkenes and stretching vibrations (Daliri et al., 2021). According to the results of the spectra, the formation of new hydrogen bonds is obvious, and the presence of phenolic extract within the maltodextrin is proved without the formation of chemical bonds.

### 3.6 XRD

X‐ray diffraction analysis is used to determine the model of bioactive encapsulation and the matrix polymorphism. The solubility rate of carriers is affected by the degree of crystallization during the transfer process (Rabbani et al., 2021). The XRD curve of pure maltodextrin (Figure 4b) showed a peak with high intensity, indicating the crystalline structure of maltodextrin. However, in the XRD curve of maltodextrin containing RGPE (Figure 4b), the intensity of the index peaks of maltodextrin decreased sharply, indicating the formation of an amorphous state of RGPE coated with maltodextrin. It shows that RGPE has been homogeneously inserted within the maltodextrin structure. That is most probably because of the high conformity of the applied material, which leads to the higher solubility of RGPE in water. Also, the amorphous state contributes to the higher loading capacity and stability of the carriers (Rabbani et al., 2021; Shahvalizadeh et al., 2021).

### 3.7 Effect of carrier and temperature on powder production efficiency

In various processes using a spray dryer, due to the use of carriers such as maltodextrin, gelatin, and other biopolymers, the glass transition temperature of the mixture increases, and the biopolymer forms a layer of non‐stick film around the feed particles. This prevents the particles from sticking to each other and also to the wall of the device (Shrestha et al., 2007). The rapid formation of the film by maltodextrin and the low moisture penetration coefficient of this film were the main factors in choosing maltodextrin as a drying agent. Maltodextrin also reduces the adhesion of particles to each other by increasing the glass transition temperature (Roos & Karel, 1991). In this study, the maltodextrin percentage in the aqueous extract of grape pulp was considered to be 30%. Adhikari et al. (2004) found that the increment in the amount of maltodextrin reduced the agglomeration and adhesion of powder particles during the storage period. Inlet air temperature and feed flow rate are other factors affecting the powder efficiency and their adhesion to each other as well as to the wall of the device (Chegini & Ghobadian, 2007). In this study, the inlet temperature used for drying was considered to be 120°C. In the early stages of drying using a co‐current spray dryer, due to the rapid contact of the feed with hot air, the process of mass and heat transfer at the particle surface is carried out at a high speed. This allows the initial stages of drying to be done very quickly. Subsequently, the surface of the particles becomes drier as the air inside the dryer gets hotter. In this study, as the temperature increased from 100 to 120°C, the powder production efficiency increased due to the reduction of semi‐dry particles adhering to each other and the wall of the drying chamber, resulting in better mass and heat transfer. At lower drying temperatures, the particles remained moist and adhered to each other and eventually to the chamber wall. On the other hand, a higher moisture content was obtained in the powder. This not only reduces the percentage of phenolic compounds but also causes the accumulation of powder due to more adhesion and therefore makes it less exposed to oxygen (Goula et al., 2004). With the temperature rising slightly above 120°C, the powder became burned or semi‐burned at the bottom of the chamber.

### 3.8 Moisture content of the powder

**FIGURE 5:**
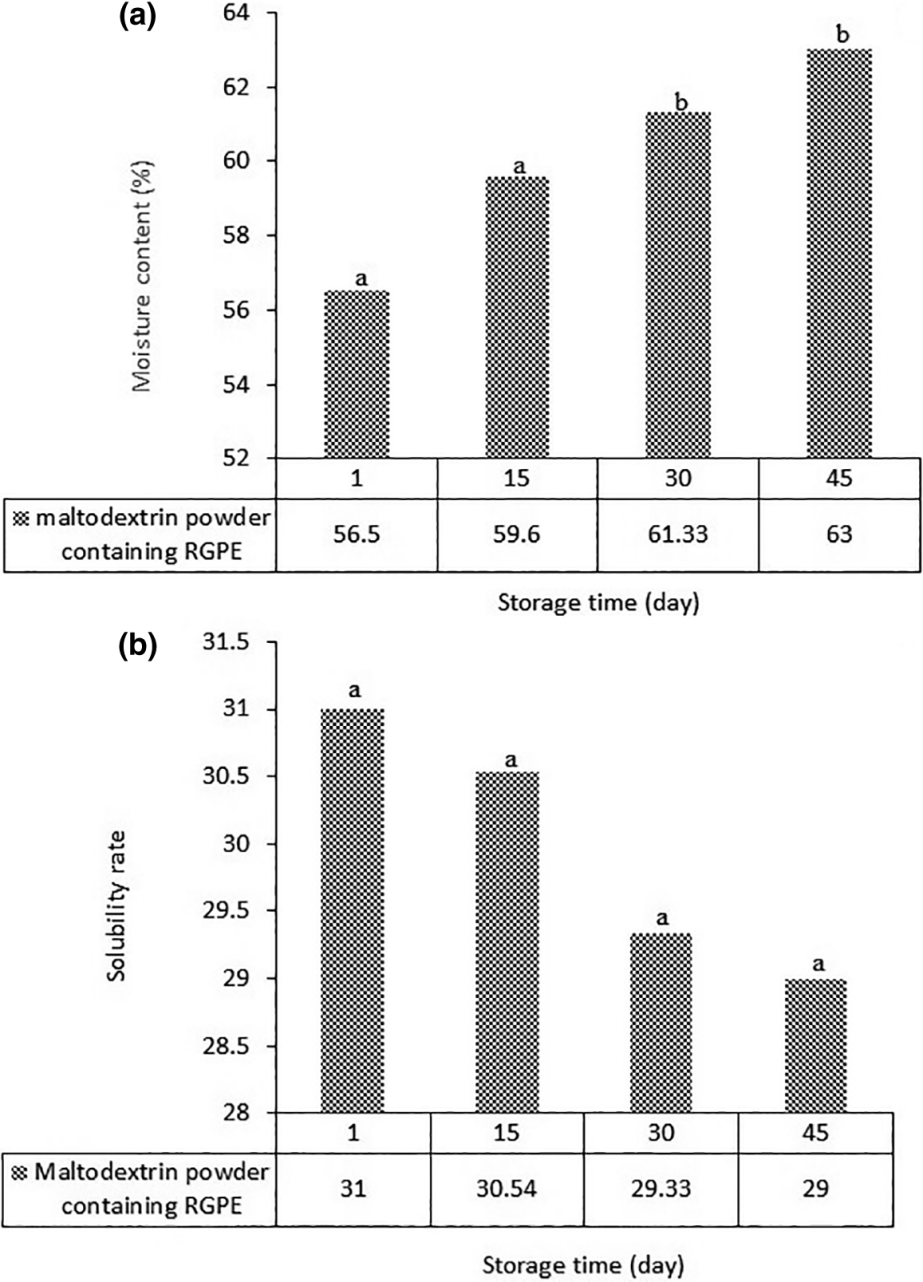
(a) Moisture content and (b) solubility rate of maltodextrin powder containing red grape pulp extract (RGPE).

As shown in Figure 5a, the moisture content of the powder was 56.5% (on the first day) and then began to increase during 45 days of storage. Factors influencing the moisture content of the final powder include the dryer temperature and the initial moisture content of the feed. The addition of maltodextrin to the feed before drying increased the solid content and also reduced the amount of evaporating water. Therefore, the powder with the desired moisture content could be produced (Abadio et al., 2004; Shahidi & Han, 1993). But if the amount of maltodextrin increases too much, the quality of the final powder decreases due to the reduction in the number of nutrients (Quek et al., 2007). Regarding the effect of temperature, the results showed that at a constant feed flow rate, the moisture content of spray‐dried powder decreased with an increase in inlet and outlet temperatures. This is because at higher inlet temperatures, the heat transfer rate is higher, providing more driving force for moisture to evaporate from the particles. As a result, a low‐moisture powder is produced (Papadakis & King, 1988). Fazaeli et al. (2012) observed a decrease in the moisture content of spray‐dried black mulberry juice powder as a result of an increase in the dryer inlet temperature. Low moisture prevents agglomeration of the resulting powder and also preserves its active ingredients and flowability properties.

### 3.9 Solubility of the powder

The solubility of powders depends on factors including the dryer temperature, the type of carrier, and the moisture content of the powder. On the 1st day, the solubility of the powder was low and then began to decrease during 45 days of storage (Figure 5b). This is probably due to the high inlet temperature, which leads to the formation of a hard surface layer around the powder particles. This phenomenon prevents the release of water molecules through the particles, reducing the solubility of the powder. These results are consistent with the results of Chegini and Ghobadian (2007). They also found that an increase in temperature reduced the solubility of the orange powder. Yousefi et al. (2011) investigated the solubility of pomegranate powder with different carriers (maltodextrin, Arabic gum, and waxy starch) and observed that the solubility of the powder was affected by the type and concentration of the carrier. According to the findings, there was also a negative relationship between the solubility and moisture content of the powder. This finding is similar to that of Goula et al. (2004).

### 3.10 The water activity of enriched gummy candy

The encapsulation of RGPE within maltodextrin by spray drying and the increment of the amount of powder in the gummy candy formulation had a significant effect on the amount of water activity. Due to the presence of maltodextrin and its hydrophilic property, the amount of water activity decreased to 0.556. Amjadi et al. (2018), in relation to the encapsulation of betanin within maltodextrin and its use in gummy candy formulation, reported that maltodextrin had a great effect on the water activity of gummy candies. In this way, the water activity of the gummy candies decreased with an increase in the amount of dry matter, reducing the amount of freezable water.

### 3.11 Antioxidant activity of enriched gummy candy

An antioxidant test was performed on the powder containing gummy candies. According to the results, the antioxidant activity of gummy candies was about 25%. Hani et al. (2015), in an experiment on fruit gummy candies containing betalain extract powder from pitaya fruit for 8 weeks, found that the higher the powder content of the gummy candy, the higher the antioxidant properties. However, the antioxidant activity of gummy candies decreased over time.

### 3.12 Color parameters of enriched gummy candy

Color is an indicator of the quality, freshness, taste status, and commercial value of the product. The L* parameter indicates the brightness, ranging from zero (black) to one hundred (full light reflection). The negative value of a* is equal to green, and the positive a* value is equal to red. In the case of the b* value, the negative value is equivalent to blue, and the positive value indicates yellow. The chroma (C*) index shows the degree of saturation and color intensity. Table 1 shows the results related to the color parameters of the control sample and the gummy candy sample enriched with maltodextrin powder containing RGPE. L* factor was higher in the control sample, indicating its brightness compared with the enriched gummy candy sample. On the other hand, the highest amount of a* value is related to the fortified gummy candy, which shows the redness of the fortified gummy candy sample. Also, both samples have positive b* values, and as a result, their color tends to be yellow. The highest amount of this factor is related to the enriched gummy candy sample, which indicates that this sample has the highest yellow color. The enriched sample has a higher hue angle compared with the control sample and therefore is yellower. The C* value of the enriched gummy candy sample is higher than the control sample, indicating the transparency of the control sample. Charoen et al. (2015), in a study on gummy candies containing a yellow extract of guajava fruit, reported that the color parameters of gummy candies decreased with the addition of the extract. Also, contrary to parameter a⃰, the two parameters L⃰ and b⃰ did not have a significant difference (in terms of the significance of the difference between the samples).

**TABLE 1:** Color parameters of different experimental samples.

Sample	*L⃰*	*a⃰*	*b⃰*	Hue angle (°)	Chroma
Control sample	60 ± 0.13ͣ	2 ± 0.21ͣ	19 ± 0.12ͣ	83 ± 0.12ͣ	19.104 ± 0.1ͣ
Enriched gummy candy	57 ± 0.1ͣ	2.5 ± 0.15ͣ	23 ± 0.151ͣ	83.795 ± 0.12ͣ	23.135 ± 0.2ͣ

*Note*: Different letters (a, b, c) in the same row indicate a statistically significant difference (*p* < .05).

### 3.13 Sensory analysis

Sensory evaluation of the experimental samples, including smell, taste, firmness, and general acceptance, is given in Table 2. The gummy candy sample containing phenolic extract powder showed acceptable sensory properties compared to the control sample, and powder addition did not cause any significant change in the parameters related to sensory evaluation. However, the best sensory characteristics were related to the control sample. Also, the results did not show much difference in terms of experimental features during 30 days of storage. These results show that the addition of powder containing phenolic extract did not cause any adverse or significant change in the organoleptic properties of the gummy candy. Also, there was no difference between the gummy candy sample containing RGPE and the control sample.

**TABLE 2:** Sensory evaluation score of odor, taste, texture (stiffness), and final acceptance of samples during 30 days of storage.

Sensory evaluation	Sample	Storage time (day)		
		1st	20th	30th
Odor	Control sample	4.1 ± 0.2^a^	4.1 ± 0.2^a^	4.3 ± 0.2^a^
	Enriched gummy candy	3.4 ± 0.2^a^	4.1 ± 0.2^a^	4.1 ± 0.2^a^
Taste	Control sample	3.6 ± 0.2^a^	4.3 ± 0.2^b^	4.1 ± 0.2^b^
	Enriched gummy candy	3.6 ± 0.2^a^	3.8 ± 0.2^a^	3.8 ± 0.2^a^
Texture	Control sample	4.1 ± 0.2^a^	4.3 ± 0.2^a^	4.1 ± 0.2^a^
	Enriched gummy candy	4.0 ± 0.0^a^	4.1 ± 0.2^ab^	4.1 ± 0.2^a^
Final acceptance	Control sample	4.1 ± 0.2^a^	4.3 ± 0.2^a^	4.1 ± 0.2^a^
	Enriched gummy candy	4.0 ± 0.0^a^	4.1 ± 0.2^a^	4.1 ± 0.2^a^

*Note*: Different letters (a, b, c) in the same row indicate a statistically significant difference (*p* < .05).

## 4 CONCLUSION

Red grape pulp extract was prepared using a green extraction method (ultrasound‐assisted extraction). Then, the obtained extract was coated with maltodextrin using a spray dryer. Finally, the produced powder was used for gummy candy enrichment. FTIR results showed that RGPE was physically loaded into the structure of maltodextrin. XRD results showed the presence of RGPE in the structure of maltodextrin without crystallization of the extract. SEM images showed the production of spherical particles of maltodextrin powder along with the placement of RGPE in its structure. The phenolic content and antioxidant activity of the extract decreased after applying the spray dryer. The encapsulation of RGPE in the structure of maltodextrin was able to maintain the antioxidant properties of RGPE in the gummy candy formulation. The sensory evaluation and color analysis of gummy candies containing RGPE powder showed acceptable results, and the addition of the RGPE powder did not cause any significant changes in the parameters related to sensory evaluation. Also, the results did not show much difference in terms of the experimental features during 30 days of storage, and from the consumer's point of view, there was no difference between the sample containing RGPE and the sample without it. Overall, the extraction of phenolic and antioxidant compounds from red grape pulp, the encapsulation of the produced extract within maltodextrin using a spray dryer, and finally its application in gummy candy enrichment could be appropriate.

## AUTHOR CONTRIBUTIONS

**Akram Pezeshki:** Project administration (lead); supervision (lead); writing – review and editing (equal). **Babak Ghanbarzadeh:** Investigation (lead); validation (lead). **Farnaz Ahmadzadeh Nobari Azar:** Writing – original draft (lead); writing – review and editing (supporting). **Hamed Hamishehkar:** Investigation (lead); resources (lead). **Maryam Heidari:** Data curation (lead); methodology (lead). **Marjan Ghorbani:** Software (equal). **Maryam Mohammadi:** Methodology (supporting); software (lead); writing – review and editing (equal).

## CONFLICT OF INTEREST STATEMENT

The authors wish to confirm that there are no known conflicts of interest associated with this publication and that there has been no significant financial support for this work that could have influenced its outcome. The authors confirm that the manuscript has been read and approved by all authors and that there are no other persons who met the criteria for authorship but are not listed. The authors further confirm that the order of authors listed in the manuscript has been approved by all of the authors. The authors confirm that they have given due consideration to the protection of intellectual property associated with this work and that there are no impediments to publication, including the timing of publication, with respect to intellectual property. In so doing, the authors confirm that they have followed the regulations of their institutions concerning intellectual property. The authors have no conflicts of interest to declare.

## Data Availability

Research data are not shared.
